# Poverty, work intensity, and disability: evidence from European countries

**DOI:** 10.1007/s10198-024-01679-x

**Published:** 2024-03-26

**Authors:** Chiara Mussida, Dario Sciulli

**Affiliations:** 1 Department of Economic and Social Sciences, Università Cattolica del Sacro Cuore, Via Emilia Parmense 84, 29122 Piacenza, Italy; 2 Department of Economic Studies, University of Chieti-Pescara, Viale Pindaro 42, 6512 Pescara, Italy

**Keywords:** Poverty, Disability, Work intensity, State dependence, Feedback effects, Panel data, J14, I32, J22, C23

## Abstract

We use 2015–2018 European Union Statistics on Income and Living Conditions longitudinal data for four European countries (the UK, Germany, France, and Italy) and a dynamic trivariate panel data model to analyze the complex relationship between poverty, work intensity, and disability. We find evidence of genuine state dependence in the three processes and feedback effects from past poverty to work intensity in all countries and from past poverty to disability in the UK, Germany, and Italy. Disability is detrimental to poverty, despite the mitigating role played by disability cash benefits. The magnitude of this effect seems to be associated with the average expenditure on social protection benefits and its distribution across functions. We stress the importance of accounting for the extra costs of disability and the key role work intensity plays in the disability–poverty connection. Finally, adopting a joint estimation strategy appears crucial to consistently estimating the relationship between the three processes.

## Introduction

According to the World Health Organization [50], 1.3 billion people, or 16% of the global population, currently have a significant disability. This number has increased in recent decades because of the aging process and the greater diffusion of chronic illness. Recent statistics reveal that in 2019, around 24% of EU people aged 16 or over experienced some limitations in daily activities (European Commission [16]). Disability, as a factor increasing vulnerability, is associated with disadvantages for individuals and related households in society and the economy. Despite the adoption of initiatives to combat this, such as the 2006 Convention on the Rights of Persons with Disabilities, the 2010 European Disability Strategy 2010–2020, and the recent EU Strategy for the Rights of Persons with Disabilities 2021–2030, equality and integration along various socioeconomic dimensions of people with disabilities is far from being reached. Descriptive statistics reveal that in 2019, disadvantages in terms of poverty for people with disabilities persisted at relatively high levels in many European countries. The risk of being poor or socially excluded in the EU-28 was over 29% for those with some or severe activity limitations and 18% for those without activity limitations, while the rates of severe material deprivation were 8.7% and 4.3%, respectively [17].

A disability can affect individual and household well-being both directly and indirectly. The need for additional goods and services implies that the rate at which income is translated into a standard of living differs between persons with and without disabilities (and their households). The literature on the extra costs of disability (e.g., [28, 43, 51]) has suggested that not accounting for the additional expenses attributable to a disability may underestimate the disadvantage of disabled people and their households [30].^1^ A full understanding of how disability influences individual and household income also requires accounting for the role of employment, which may affect the formation of household income. People with disabilities are less likely to be employed (e.g., [18, 37]), and a person with disabilities may affect the employment probabilities of other household members (especially women) if caregiving activities are provided informally by the family unit (e.g., [8, 38]).

The existing literature has offered partial insights into these aspects. One stream has highlighted the income disadvantage of people with disabilities, through analyses of the disability–poverty relationship (e.g., [22] for the UK [19], for Ireland [38], for Italy [14], for Spain [26], for the US [47], for Cambodia). Moreover, there is a vast literature discussing other forms of poverty that may be associated with disability, such as multidimensional poverty [12, 30, 39], or economic disadvantage stemming from poor employment outcomes of people with disabilities (e.g., [18, 23, 37]) or their family carers (e.g., [8, 35]).

However, no studies have analyzed the link between disability, employment, and income conditions in a structural way, despite these all potentially being correlated. This link somewhat depends on the dynamic nature of the poverty–employment–disability relationship, which determines potential endogeneity problems if not accounted for. Indeed, current employment and disability status may be predetermined by previous income conditions. The former, for example, relates to poor individuals/households usually being associated with lower skills and educational attainment and, thus, lower chances of being employed. The latter is because the poverty condition is usually associated with a lower health status, due to difficulties in accessing the health care system, which may lead to a faster deterioration of health. These aspects have been considered in a few studies (e.g., [19, 22, 39]) that are essentially limited to the disability–income relationship, with employment being treated as exogenous.

This study contributes to the literature in various ways. First, we model the disability–poverty relationship by incorporating the role of employment outcomes in a unique framework. In contrast to previous contributions, this allows explicitly modeling all processes and considering their intrinsic dynamic correlations. Linked to the adoption of such a framework, the second contribution is the possibility of considering the endogeneity of employment and disability in the poverty process through feedback effects (e.g., [22]), i.e., the possibility that past poverty may be detrimental to current employment and disability status.^2^ This is important from an economic point of view, because the presence of feedback effects would suggest that reducing poverty would determine positive spillovers for employment and disability in the medium–long term. Finally, we offer a multi-country analysis, which allows us to highlight the role of the social protection systems of the analyzed countries in affecting the relationship between poverty, employment, and disability.

The analysis is based on longitudinal European Union Statistics on Income and Living Conditions (EU-SILC) data spanning 2015–2018 for four major Western European countries, i.e., the UK, Germany, France, and Italy. These countries are relevant in terms of size, representing around half of the European population. They are also characterized by different poverty rates, work intensity, and shares of people with disabilities,^3^ as well as different social protection systems, which may contribute to determining distinct relationships between the investigated processes.

Our empirical strategy consists of adopting a dynamic trivariate panel data model, in line with Biewen [6] and Ayllón [3]. This enables us to account for state dependence, random effects, and endogenous initial conditions, thus allowing the consistent estimation of state dependence in the three processes. The model explicitly allows for feedback effects and correlation between each equation's random effects and the error term. We explicitly consider the extra costs of disability by adopting a disability-corrected equivalence scale (i.e., [24]) to properly weigh the presence of people with disabilities in a household (see Sect. “Data and variables”). Finally, we approximate employment at the household level using the work-intensity indicator (see [12]).

Our results show the existence of sizeable genuine state dependence in all investigated processes and the presence of feedback effects from past poverty to work intensity and from past work intensity to disability. Feedback effects from past poverty to disability are observed in the UK, Germany, and Italy. We find evidence of the detrimental effects of disability on poverty in all countries, despite the mitigating role played by disability cash benefits. The negative impact, however, is smaller in countries with relatively high levels of disability benefits (especially in-kind transfers), and where reconciliation policies and a more inclusive labor market for females are better promoted.

The paper is organized as follows. The section “The literature” reviews the existing literature. The section “Data and variables” presents the dataset and provides descriptive statistics. The empirical model is described in the section “The econometric approach”. The section “Results” discusses the main findings, and the section “Concluding remarks” offers some concluding remarks.

## The literature

The literature analyzing the living standards of people with disabilities and their households has broadened in the last 2 decades. There is a stream on the relationship between disability and income poverty, which is one of the indicators used to monitor social exclusion, together with severe material deprivation and very low work intensity (e.g., [22] for the UK [19], for Ireland [38], for Italy [45], for the US [14], for Spain [26], for the US [47], and for Cambodia).

Jenkins and Rigg [22] analyzed the economic disadvantage suffered by working-age persons with disabilities in the UK. They stressed that this disadvantage is guided by three different processes, i.e., pre-existing disadvantage, the onset of disability, and the duration of disability. The existence of a selection effect into a disability because of one’s past poverty condition is also stressed by Gannon and Nolan [19] for Ireland, who argued that having been in a low-income household in the previous year increases the probability of disability onset. They also found that both persistent disability and disability onset determine a lower chance of being socially included in terms of poverty and social participation.

Parodi and Sciulli [38] showed that disability increases the risk of being poor in Italian households, mainly due to both the inadequacy of public policies in fully supporting disability and the negative effects on the labor supply of household members providing informal care. She and Livermore [45] confirmed that working-age adults with disabilities in the US experience a higher poverty rate than people without disabilities, especially in the long term. Davila-Quintana and Malo [14] found that past disability slightly increases the current risk of being poor for Spanish households and that this effect increases in the long term. Again for the US, Meyer and Mok [26] found that the onset of severe and chronic disability increases the risk of poverty, but income conditions tend to improve in the long run. Takasaki [47] found that disability onset causes reduced work, earnings, and income and increases poverty among amputees in rural Cambodia.

As mentioned in the Introduction, there is a vast and growing literature discussing other forms of poverty that may be associated with disability, such as multidimensional poverty. This includes studies on developing countries, in particular (e.g., [4, 27, 29, 41, 48]). Recently, Mitra and Palmer [30] reviewed the literature that documents and explains differences in economic well-being—that is, poverty measured through monetary and multidimensional measures—based on disability status, considering low- and middle-income countries. There is also a growing literature on high-income countries (see [10] for the US [46], for European countries, and [39] and [12] for Italy).

Another strand of literature has investigated the disability–employment relationship by considering economic disadvantage as a consequence of the poor employment outcomes of people with disabilities (e.g., [18] for Ireland, [37] for Canada, and [23] for the UK) or their family carers (e.g., [8] for Germany and [35] for Italy, France, and the UK).

Nonetheless, the dynamic relationships between the phenomena of disability, employment, income poverty, and potential endogeneity problems have not been fully explored. A few studies dealing with these aspects (e.g., [19, 22, 39]) are essentially limited to the disability–income relationship, with employment being treated as exogenous. We aim to fill this gap in the literature by offering novel evidence for four major Western European countries.

Finally, we account for the extra costs of disability, i.e., the need for special/additional goods and services for individuals with disabilities. The literature on the extra costs of disability (e.g., [13, 28, 31, 32, 43, 51]) suggests that once this is accounted for, disability may be particularly detrimental for income poverty.

## Data and variables

### Data and sample

We use data from the 2015–2018 longitudinal sample of the European Union Statistics on Income and Living Conditions (EU-SILC) survey. The survey is conducted in most European Union countries by the relevant national institutes of statistics, using harmonized definitions and survey methodologies. The survey covers living conditions, income, social exclusion, housing, work, demography, and education. EU-SILC is a rotating panel survey: the sample for any given year consists of four replications that have taken part in the survey for 1–4 years. Each replication remains in the survey for 4 years; each year, one of the four replications from the previous year is dropped and a new one is added. Between years *T* and *T* + 1, the sample overlap is 75%; the overlap between year *T* and year *T* + 2 is 50%; and it is reduced to 25% from year *T* to year *T* + 3 and to 0 for longer intervals.

The sample we analyze is the result of a complex design. The relevant published sampling weights account for the unequal inclusion probabilities along with the effect of non-response and panel attrition. We do not use these weights in regression analyses. The main reason is that we have access to all relevant sampling design information (stratum and cluster identifiers). In addition, our implicit assumption that the sampling design is non-informative given the covariates has been tested according to the procedure illustrated in Pfeffermann and Sverchkov [40], with the null hypothesis not being rejected.

We analyze data from four major Western European countries: the UK, Germany, France, and Italy. We selected these countries as they are relevant in terms of size, representing around half of the European population. Moreover, they are characterized by different poverty rates, work intensities, and shares of people with disabilities, as well as different institutional settings, welfare regimes, and policies.

We focus on the relationships among the phenomena of at-risk-of-poverty, household work intensity, and disability/limitations in activities, and our units of analysis are the individuals. We consider the fraction of the sample for which *T* ≥ 3, which refers to 3 or 4 consecutive years. Furthermore, we explore samples of household with at least one individual aged below 60 years, as this allows the calculation of the indicator for work intensity, as we will see in the next section. This selection leaves us with 20,422 observations for the UK, 20,323 for Germany, 32,385 for France, and 39,231 for Italy for the 2015–2018 period.

### Outcomes and covariates

**Fig.1:**
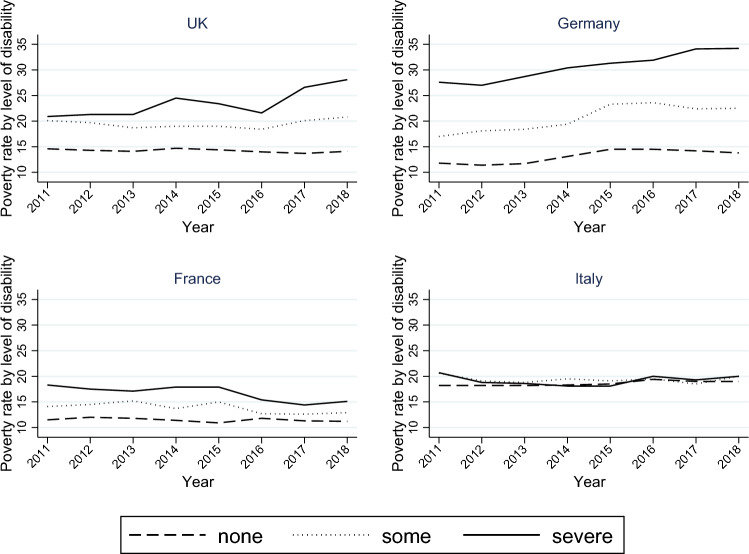
Poverty rates by level of disability. Source: eurostat statistics

In what follows, we describe the main variables used in our analysis and how these are operationalized. We consider three dependent variables (for three equations; details can be found in Sect. “The econometric approach”): at-risk-of-poverty, household work intensity, and disability/limitations in daily activities. At-risk-of-poverty is defined at the individual level and refers to people whose equivalent income is less than 60% of the equivalent national median. Equivalized income is the total disposable household income (after taxes and social transfers) divided by an equivalence scale that gives weight to each person in the household. For households without disabled members and with members with some disabilities, the scale coincides with the modified OECD scale, whereas, in line with Davila-Quintana and Malo [14], for households with severely disabled members we use the disability-corrected equivalence scale suggested by Kuklys [24].^4^ The poverty rates by level of disability for all countries explored are shown in Fig. 1.

We see that the poverty rates differ significantly across the countries explored as well as between different levels of disability. This heterogeneity across countries and levels of disability might be (at least partly) due to differences in institutions and social policies.

While most studies that analyze the effect of disability on labor market participation rely on the categorical labor market status of employment [21], an increasing number of works (i.e., [9]) highlight that this indicator offers only a partial view of the labor market, especially in contexts characterized by high flexibility. Therefore, to better disentangle labor market attainment, we use a more flexible measure of labor market participation: work intensity. Work intensity is calculated at the household level as “the number of months that all working-age household members have been working during the income reference year as a proportion of the total number of months that could theoretically be worked within the household.”^5^ The indicator is calculated for adults aged 18–59 but excluding students from the age of 18 to 24. In this study, we follow the EU-SILC survey and define work intensity level through four categories (variable HX020 in the EU-SILC questionnaire): 0, (0, 0.5), [0.5, 1), and 1. Work intensity is particularly suitable for capturing labor market attachment in the presence of disability as it represents a unitary measure of household labor supply and accounts for different levels of labor-supply intensity due, for instance, to full-time vs. part-time employment of working-age household members. Indeed, we extend the Eurostat definition of work intensity to account for part-time employment by assuming that this is equivalent to (approximately) 50% of full-time employment.

As for disability, the EU-SILC survey (variable PH030) provides information on disability status based on a question about limitations in daily activities that arise due to health problems: “Has the respondent experienced limitations in daily activities due to health problems for at least the last six months?” Respondents were asked to assess their own limitations (perceived disability) by choosing between three levels of severity: 1. Yes, severely limited; 2. Yes, limited; 3. No, not limited. This variable enables us to distinguish among individuals with severe activity limitations, those with some limitations, and those with no limitations due to health problems, and therefore to identify different severity levels of disability. The identification of disability through activity limitation allows us to consider disability as the result of the interaction between impairment and the external environment, in the spirit of the social model of disability.

We extend the individual information on disability to all household members. This allows us to qualify the disability variable as an indicator of living in a household characterized by the presence of a member with a disability. The rationale relates to considering disability as a household-level problem in light of the shared experiences of household members in terms of caring activities and the lower marginal utility of income because of extra costs. A possible limitation of the adopted definition of disability is related to the subjective nature of the self-reported information through which disability is identified. The use of self-reporting can potentially be affected by justification bias, as individuals may exaggerate their limitations to justify poor income conditions and/or poorer performance in the labor market (e.g., [5]). However, the latter effect may be mitigated, because we refer to household work intensity rather than the individual labor supply of persons with disabilities. In addition, dealing with feedback effects from past poverty to disability may help to alleviate bias determined by justifying behaviors. One also has to consider that answers to disability questions in survey data may be affected by different understandings of the phenomenon across individuals and cultures. Mitra et al. [29], for instance, suggest that individuals may report only extreme cases of impairment, due to stigma or misperception of the phenomenon among older people. An alternative way to define disability could be to use information on the receipt of disability benefits in the EU-SILC data (e.g., [38]). A potential advantage would be the greater objectivity of the information. However, this approach may suffer from severe bias, because the probability of applying for and/or accessing disability benefits may crucially depend on individual characteristics, such as education and age, or differences in eligibility criteria across countries.

Having defined the main variables of the study, we again stress that the unit of analysis is the individual, as suggested by the standard approach to poverty investigation. Starting from disposable household income, indeed, equivalent income is attributed to each member of the related household, and poverty is defined on an individual basis. For consistency, we follow the same strategy for work intensity and disability. Thus, for each individual, the variable indicates whether she lives in a household with different levels of work intensity and a household characterized by the presence of a (moderately or severely) disabled member.

We now briefly describe the covariates used, keeping in mind that due to the frameworks employed, we also include lagged dependent variables and initial conditions, as well as the average and initial values of time-varying covariates. Relevant descriptive statistics are reported in Table 1.

**Table 1:** Descriptive statistics

Variable	UK		Germany		France		Italy	
	Mean	Std. dev	Mean	Std. dev	Mean	Std. dev	Mean	Std. dev
Dependent variables								
Poverty	0.154	0.361	0.134	0.341	0.130	0.336	0.183	0.387
Work intensity								
0	0.123	0.328	0.104	0.306	0.082	0.275	0.133	0.340
(0, 0.5)	0.071	0.256	0.074	0.262	0.097	0.296	0.139	0.346
[0.5, 1)	0.454	0.498	0.475	0.499	0.421	0.494	0.386	0.487
1	0.353	0.478	0.347	0.476	0.399	0.490	0.341	0.474
Disability								
Not disabled	0.603	0.489	0.689	0.463	0.661	0.473	0.704	0.457
Some limitations	0.206	0.404	0.206	0.404	0.214	0.410	0.217	0.412
Severe limitations	0.191	0.393	0.105	0.306	0.125	0.331	0.079	0.270
Covariates								
HH aged less than 25	0.040	0.196	0.028	0.165	0.031	0.174	0.019	0.137
HH aged 25–34	0.153	0.360	0.126	0.332	0.146	0.354	0.121	0.326
HH aged 35–44	0.250	0.433	0.197	0.398	0.240	0.427	0.230	0.421
HH aged 45–54	0.320	0.467	0.346	0.476	0.344	0.475	0.338	0.473
HH aged 55–64	0.199	0.399	0.272	0.445	0.222	0.416	0.241	0.428
HH aged more than 64	0.038	0.191	0.031	0.172	0.016	0.124	0.051	0.220
HH female	0.360	0.480	0.341	0.474	0.354	0.478	0.319	0.466
HH low educated	0.208	0.406	0.068	0.251	0.170	0.376	0.329	0.470
HH middle educated	0.306	0.461	0.520	0.500	0.458	0.498	0.464	0.499
HH highly educated	0.486	0.500	0.412	0.492	0.371	0.483	0.207	0.405
HH married	0.709	0.454	0.690	0.462	0.753	0.431	0.622	0.485
Disabled individual of working age	0.105	0.306	0.081	0.272	0.073	0.259	0.148	0.356
Disabled individual with low education	0.184	0.388	0.093	0.291	0.175	0.380	0.216	0.412
Disabled female individual	0.367	0.482	0.289	0.453	0.317	0.465	0.281	0.450
Children aged 0–15	0.414	0.493	0.275	0.447	0.409	0.492	0.296	0.456
Homeowner	0.692	0.462	0.538	0.499	0.675	0.468	0.731	0.443
Disability Benefit *t* − 1	0.138	0.345	0.070	0.256	0.044	0.206	0.076	0.264
Observations	20,422		20,323		32,385		39,231	

Mean (percentages) and standard deviations

Source: Authors’ elaborations on EU-SILC data

Our control variables can be classified into individual and household characteristics. The individual characteristics refer to the characteristics of the head of household and include age (splitting into age ranges from less than 25 years to more than 64 years), gender, education, and marital status. Moreover, we include individual controls in the disability equation: a dummy variable for disabled individuals of working age (1 if the disabled person is aged 16–60, 0 otherwise), one for disabled individuals with low education (1 if the disabled person is educated only to the primary level, 0 otherwise), and another indicating the gender of the disabled individual (1 if female, 0 otherwise). The household characteristics include controls for the presence of children aged from 0 to 15 years and for home ownership. We also control for the receipt of disability cash benefits (variable PY130G in the EU-SILC code) in the previous year. We consider the lag of the variable to mitigate the risk of reverse causality. The variable is included in all equations to assess the role of disability policies.

From Table 1, we see that the provision of disability cash benefits is relatively low in all countries explored, ranging from 4.4% in France to 13.8% in the UK. Finally, in our models estimated separately by country, we control for macro-region and time dummies.

## The econometric approach

The analysis is based on a first-order Markov random-effects trivariate model.^6^ This enables us to estimate the degree of state dependence for each phenomenon and to account for possible endogeneity because of feedback effects and selection issues related to shared unobservable factors. This allows us to relax the strict exogeneity assumption, which is important in our context as the dynamics of poverty, work, and disability may be intrinsically correlated. We extend the trivariate probit method adopted by Biewen [6] and Ayllòn [3] to study poverty dynamics and its determinants, modeling poverty as a dichotomous outcome and work intensity, and disability as ordinal outcomes. The model has a recursive structure, implying that the three equations are not simultaneous, i.e., current poverty does not enter the work intensity and disability equations. Biewen [6] has indeed stressed that simultaneous systems of qualitative outcomes are logically inconsistent. From an economic point of view, this choice reflects the assumption that current disability affects poverty via work intensity. This appears plausible as current disability determines immediate effects on poverty by influencing, for example, the labor supply of people with disabilities. However, the deterioration of health conditions because of low-income levels is likely to require a longer time, thus justifying the hypothesis that current disability is affected by past poverty but not current poverty. The same applies to the disability–work intensity and the work intensity–poverty relationships.

Let us define *p*_*it*_ as the (disability-adjusted) poverty status, *wi*_*it*_ as the (ordinal) work intensity status, and *d*_*it*_ as the (ordinal) disability status, where *i* = 1*…N* are the individuals and *t* = 1*…T* refers to the analyzed years. The related latent propensity of each outcome is defined in Eqs. 1, 3, and 5, respectively. The latent poverty propensity ${p}_{it}^{*}$ reads as

1 $${p}_{it}^{*}={\alpha }_{1}{p}_{it-1}+{\alpha }_{2}{wi}_{it}+{\alpha }_{3}{w}_{iit-1}+{\alpha }_{4}{d}_{it}+{\alpha }_{5}{d}_{it-1}+{\alpha }_{6}{x}_{it}+{\alpha }_{7}{z}_{i}+{a}_{i}+{u}_{it}.$$

The unobserved latent poverty propensity variable has a corresponding observable binary variable, which identifies the poverty outcome and can be expressed as follows:

2 $$p_{{{\text{it}}}} = \left\{ {\begin{array}{*{20}c} {1\quad {\text{if}}\quad p_{it}^{*} > 0,} \\ {0 \quad {\text{otherwise}}. } \\ \end{array} } \right.$$

Equation 3 specifies the unobserved latent work intensity propensity ${wi}_{it}^{*}$

3 $${wi}_{it}^{*}={\beta }_{1}{p}_{it-1}+{\beta }_{2}{wi}_{it-1}+{\beta }_{3}{d}_{it}+{\beta }_{4}{d}_{it-1}+{\beta }_{5}{x}_{it}+{\beta }_{6}{z}_{i}+{h}_{i}+{v}_{it.}$$

The corresponding observed ordinal variable identifying the work intensity outcome (which ranges from 0 to 1) reads as

4 $$wi_{it} = \left\{ {\begin{array}{*{20}c} {0\quad {\text{if}} \quad wi_{it}^{*} \le c_{1} ,} \\ {1{ }\quad {\text{if}}\quad c_{1} < wi_{it}^{*} \le c_{2} ,} \\ { 2 { }\quad {\text{if}} \quad c_{2} < wi_{it}^{*} \le c_{3} ,} \\ {3\quad {\text{if}}{\kern 1pt} \quad wi_{it}^{*} > c_{3} ,} \\ \end{array} } \right.$$

where *wi* = 0 indicates zero work intensity, *wi* = 1 indicates work intensity greater than zero and less than 0.5, *wi* = 2 indicates work intensity equal to 0.5 or greater but less than 1, and *wi* = 3 indicates full work intensity. The latent disability propensity ${d}_{it}^{*}$ takes the following form:

5 $${d}_{it}^{*}={\gamma }_{1}{p}_{it-1}+{\gamma }_{2}{wi}_{it-1}+{\gamma }_{3}{d}_{it-1}+{\gamma }_{4}{x}_{it}+{\gamma }_{5}{z}_{i}+{\gamma }_{6}{r}_{it}+{g}_{i}+{\varepsilon }_{it.}$$

Finally, the observed ordinal variable identifying the disability outcome reads as

6 $$d_{it} = \left\{ {\begin{array}{*{20}c} {0\quad if\quad d_{it}^{*} \le k_{1} ,} \\ {1 \quad if\quad k_{1} < d_{it}^{*} \le k_{2} ,} \\ {2\quad if\quad d_{it}^{*} > k_{2.} ,} \\ \end{array} } \right.$$

where *d* = 0 indicates no disability, *d* = 1 indicates moderate disability, and *d* = 2 indicates severe disability.

In Eqs. (1), (3), and (5), *p*_*it-*1_ is the lagged (disability-adjusted) poverty status,* wi*_*it-*1_ is the lagged work intensity status, and d_*it-*1_ is the lagged disability status, while *x*_*it*_ and *z*_*i*_ are vectors of strictly exogenous time-variant and time-invariant (respectively) individual and household characteristics. The former includes the lagged disability benefit variable. *r*_*it*_ is a vector of strictly exogenous characteristics of household members with a disability (Eq. 5). *α*_1_ is the poverty state-dependence parameter, while *β*_2_ and *γ*_3_ are vectors of work intensity and disability state-dependence parameters, respectively. The parameters *β*_1_ and *γ*_1_ recognize the presence of feedback effects from poverty to work intensity and disability, while *γ*_2_ identifies the potential feedback effect from work intensity to disability. *α*_2_, *α*_3_, *α*_4_, *α*_5_, *α*_6_, *α*_7_, *β*_3_, *β*_4_, *β*_5_, *β*_6_, *γ*_4_*,* and *γ*_5_ are sets of parameters to be estimated. The terms *a*_*i*_, *h*_*i*_*,* and *g*_*i*_ represent the unobserved time-invariant individual effects for the analyzed processes, while *u*_*it*_, *v*_*it*_*,* and *ε*_*it*_ are the idiosyncratic error terms for poverty, work intensity, and disability phenomena, which we assume to be normally distributed with zero mean and unit variance and not serially correlated (see [6]). This means that our trivariate model corresponds to a probit—ordered probit—ordered probit random-effects model. Finally, *c*_1_, *c*_2_, *c*_3_, *k*_1_*,* and *k*_2_ are threshold parameters to be estimated.

When estimating a dynamic random-effects model, the presence of unobserved heterogeneity poses at least two issues to be solved, i.e., the initial conditions problem [21] and the incidental parameters problem. The former is dealt with using the Wooldridge approach [49], which involves the use of an alternative conditional maximum likelihood (CML) estimator that considers the distribution conditional on the value in the initial period. The latter implies that the time-invariant unobserved individual effects cannot be considered as standard parameters to be estimated. Thus, we relax the assumption that individual-specific random effects are independent of other covariates by assuming correlated random effects [34]. According to Wooldridge [49], the conditional density of the unobserved effects *a*_*i*_, *h*_*i*_, and *g*_*i*_ are specified via auxiliary models. Building on the debate on short-panel issues launched by Akay [1], Rabe-Hesketh and Skrondal [42] have suggested extending Wooldridge’s approach [49] by including as additional regressors the initial period of time-varying variables in the auxiliary models, with the aim of reducing the substantial finite-sample bias. Our analysis relies on the Rabe-Hesketh and Skrondal [42] solution, and thus, the auxiliary models definitively read as

7 $${a}_{i}={\theta }_{0}+{\theta }_{1}{p}_{i1}+{\theta }_{2}{wi}_{i1}+{\theta }_{3}{d}_{i1}+{\theta }_{4}{\overline{x} }_{i}+{\theta }_{5}{x}_{i1}+{\mu }_{i,}$$

8 $${h}_{i}={\pi }_{0}+{\pi }_{1}{p}_{i1}+{\pi }_{2}{wi}_{i1}+{\pi }_{3}{d}_{i1}+{\pi }_{4}{\overline{x} }_{i}+{\pi }_{5}{x}_{i1}+{\epsilon }_{i},$$

9 $${g}_{i}={\delta }_{0}+{\delta }_{1}{p}_{i1}+{\delta }_{2}{wi}_{i1}+{\delta }_{3}{d}_{i1}+{\delta }_{4}{\overline{x} }_{i}+{\delta }_{5}{x}_{i1}{+\delta }_{6}{\overline{r} }_{i}+{\delta }_{7}{r}_{i1}+{\kappa }_{i},$$

where *p*_*i*1_ is the initial (disability-adjusted) poverty status, *wi*_*i*1_ is the initial work intensity status, and *d*_*i*1_ is the disability status at time 1. ${\overline{x} }_{i}$ and ${\overline{r} }_{i}$ are sets of time-averaged time-variant control variables calculated from periods 2 to T, while *x*_*i*1*\*_ and *r*_*i*1_ are sets of initial values of time-varying covariates. *θ*_*k*_, *π*_*k*_*,* and *κ*_*k*_ are sets of parameters to be estimated. Finally, the terms ${\mu }_{i}$, ${\epsilon }_{i}$, and ${\kappa }_{i}$ are the residual unobserved heterogeneity, which is assumed to be independent of observed characteristics. We assume that unobservable individual factors are drawn from a trivariate normal distribution with zero mean and variance ${\sigma }^{2}$, and their association is captured by the following correlation terms: ${\rho }_{pw}={\text{corr}}\left({\mu }_{i},{\epsilon }_{i}\right)$, ${\rho }_{pd}={\text{corr}}\left({\mu }_{ic},{\kappa }_{ic}\right),$ and ${\rho }_{wd}={\text{corr}}\left({\epsilon }_{ic},{\kappa }_{ic}\right)$, where each *ρ* term represents the two-by-two correlation between unobservable factors of the outcomes considered. As stressed by Ayllón [3],^7^ whether the correlation terms are statistically significant or not is important to assess the opportunity to use a joint estimation approach. In particular, if the correlation terms are statistically different from zero, the unitary three-equation model is crucial to avoid inconsistent estimates. Finally, we compute and report the average marginal effects (AMEs) to appropriately determine the magnitude of the impact between outcome and explanatory variables.

## Results

In the following, we comment on the AMEs for the variables of interest by country. In the section “Poverty”, we examine the poverty equation, while in the sections “Work intensity” and “Disability”, we present the results of the work intensity (WI) and disability equations, respectively.^8^ For work intensity, we focus on full work intensity, while for disability we focus on severe activity limitations.^9^ We discuss supplementary analyses in the section “Further results and supplementary analyses”. Finally, we reflect on the cross-country differences in the poverty–employment–disability relationship in the section “Reflections on cross-country differences”.

### Poverty

Table 2 reports the AMEs of interest for the poverty equation. In the three panels, we see the effect of state dependence and the initial condition of poverty, the relationship between WI and poverty, and the association between disability and poverty. As expected, poverty status at *t*–1 is positive and significant in each of the analyzed countries, and this signals the existence of genuine state dependence in income poverty: being poor today in itself increases the chances of being poor tomorrow. The AMEs in Table 2 indicate that genuine state dependence in poverty varies from 3.6 to 7.4 percentage points (pp.), with the lowest found in Germany and the highest in Italy. The significance of the parameters for initial poverty status suggests that unobserved heterogeneity is a relevant issue in all countries explored.

**Table 2:** Poverty equation

			UK		Germany		France		Italy	
			AME	s.e	AME	s.e	AME	s.e	AME	s.e
Poverty	Time *t* − 1		0.050	0.011***	0.036	0.009***	0.047	0.007***	0.074	0.009***
	Time 1		0.115	0.008***	0.109	0.007***	0.090	0.005***	0.146	0.005***
Work intensity	Time *t*	0 < WI ≤ 0.5	− 0.035	0.038	0.000	0.011	− 0.037	0.038	− 0.010	0.018
		0.5 < WI < 1	− 0.078	0.065	− 0.002	0.017	− 0.075	0.063	− 0.081	0.033**
		WI = 1	− 0.095	0.089	0.032	0.033	− 0.086	0.083	− 0.117	0.045***
	Time *t* − 1	0 < WI ≤ 0.5	0.014	0.017	− 0.007	0.016	− 0.021	0.014	0.013	0.008
		0.5 < WI < 1	− 0.006	0.021	− 0.027	0.019	− 0.029	0.023	− 0.005	0.010
		WI = 1	− 0.008	0.032	− 0.051	0.025**	− 0.036	0.035	− 0.005	0.015
	Time 1	0 < WI ≤ 0.5	− 0.016	0.022	− 0.063	0.022***	0.016	0.009*	− 0.016	0.009*
		0.5 < WI < 1	− 0.041	0.024*	− 0.069	0.024***	0.013	0.010	− 0.014	0.010
		WI = 1	− 0.062	0.031**	− 0.087	0.030***	0.006	0.014	0.001	0.014
Disability	Time *t*	Disability	0.009	0.006	0.003	0.005	0.004	0.004	− 0.001	0.004
		Severe disability	0.140	0.019***	0.103	0.023***	0.111	0.014***	0.122	0.018***
	Time *t* − 1	Disability	− 0.008	0.007	− 0.002	0.006	0.005	0.004	− 0.005	0.004
		Severe disability	− 0.015	0.009	− 0.008	0.009	− 0.002	0.006	− 0.051	0.007***
	Time 1	Disability	0.006	0.008	0.006	0.007	− 0.006	0.005	− 0.008	0.005
		Severe disability	− 0.010	0.010	− 0.003	0.010	− 0.018	0.006***	− 0.024	0.007***
	Time *t* − 1	Benefit	− 0.040	0.010***	− 0.005	0.007	− 0.029	0.008***	− 0.030	0.006***

Source: Authors’ elaborations on EU-SILC data

We control for the set of covariates described in the section “Data and variables” including regional and year dummies. *p < .10, **p < .05, ***p < .01

From the second panel of Table 2, we note that current WI is only associated with poverty in Italy. In this country, current labor market attachment reduces the risk of poverty, especially for full work intensity. This highlights the important role that labor market outcomes play in mitigating poverty, especially in Southern Europe. Despite being not significant, we note that in the UK and France, the magnitude of the effect is not negligible, due to the high variability of the estimated effect. For Germany, instead, the effect is both not significant and negligible, likely as a result of the protective role against poverty played by the transfer system for the working-age population (i.e., [11]). Notably, the importance of work intensity to combat at-risk-of-poverty appears in the medium–long term for all countries, and especially the UK and Germany, again due to the presence of protections and transfers that mitigate the risk of poverty.

In the third panel of Table 2, we investigate the relationship between disability and income poverty by disentangling the role of moderate and severe limitations in daily activities. Despite accounting for the extra costs of disability using the disability-adjusted equivalence scale, current severe disability is positively and strongly associated with poverty. The AMEs are relatively consistent across countries, ranging from + 10.3 pp. for Germany to + 14 pp. for the UK (the AME for France is 11.1 pp. and 12.2 pp. for Italy). In general, these results suggest that severe disability is detrimental to income conditions and that income-support mechanisms for households with disabled members might not be able to fill the gaps between the analyzed subgroups of the population. In addition, we find sparse evidence of statistically significant effects in the medium term. In particular, in France the presence of a severely disabled person at time 1 decreases the risk of poverty by 1.8 p.p. In Italy, the presence of a severely disabled person at time *t*–1 decreases the risk of poverty by 5.1 p.p., whereas this decreases to 2.4 p.p. when referring to time 1. For the UK and Germany, AMEs are negative but small and statistically not significant. Several non-exclusive causes may explain these negative associations. First, social support may need time to exert its mitigating effects. Second, in the medium term, households may adopt effective strategies to cope with severe disability conditions, thus determining a recovery of income status. In this regard, the literature suggests the possibility of a recovery in various economic outcomes (such as income and consumption) some years after a disability shock (e.g., [26]).

Finally, we look at the possible role of past (previous year) disability cash benefits on poverty. This association is quite important as it might indicate the effectiveness—or lack thereof—of disability (and related) benefit provision on the alleviation of poverty [for details on the relative level of social protection benefits (total, cash, and in-kind by country), see Table 6]. From the last row of Table 2, we see that the presence of disability cash benefits in the previous year is negatively associated with current poverty in all countries, with the exception of Germany. This is a sign of the efficacy of the cash component of disability benefits in alleviating at-risk-of-poverty (e.g., [25]. Notably, the lack of significance for the cash component of disability benefits in Germany might be compensated by the provision of in-kind benefits (see Table 6).

### Work intensity

Table 3 reports the AMEs for the work intensity equation. This equation also includes lagged poverty experience, capturing possible feedback effects from poverty to work intensity. Such feedback effects may reflect the detrimental effects of poverty on the morale of individuals, leading to fewer employment opportunities. Moreover, for some individuals, the experience of poverty or, more generally, a feeling of social exclusion might lead to problems with alcohol and/or drugs and hence to health problems, which typically make it difficult to return to better-paid employment positions or even to remain employed [6].

**Table 3:** Work intensity equation: outcome WI = 1

			UK		Germany		France		Italy	
			AME	s.e	AME	s.e	AME	s.e	AME	s.e
Poverty	Time *t* − 1		− 0.057	0.013***	− 0.002	0.013	− 0.055	0.010***	− 0.015	0.010
	Time 1		− 0.012	0.012	− 0.051	0.013***	− 0.019	0.009**	− 0.028	0.009***
Work intensity	Time *t* − 1	0 < WI ≤ 0.5	0.051	0.009***	0.063	0.011***	0.038	0.005***	0.043	0.007***
		0.5 < WI < 1	0.190	0.008***	0.213	0.008***	0.202	0.007***	0.169	0.008***
		WI = 1	0.496	0.019***	0.531	0.024***	0.680	0.014***	0.492	0.022***
	Time 1	0 < WI ≤ 0.5	0.116	0.016***	0.072	0.016***	0.103	0.015***	0.060	0.010***
		0.5 < WI < 1	0.185	0.012***	0.181	0.014***	0.173	0.016***	0.153	0.011***
		WI = 1	0.386	0.023***	0.458	0.032***	0.334	0.026***	0.337	0.024***
Disability	Time *t*	Disability	− 0.017	0.007**	− 0.029	0.007***	− 0.004	0.005	− 0.015	0.004***
		Severe disability	− 0.045	0.013***	− 0.034	0.015**	− 0.056	0.011***	− 0.064	0.010***
	Time *t* − 1	Disability	0.004	0.008	0.000	0.008	− 0.011	0.006*	0.005	0.005
		Severe disability	0.008	0.012	− 0.035	0.015**	− 0.006	0.010	− 0.011	0.009
	Time 1	Disability	0.003	0.009	0.002	0.009	0.000	0.006	− 0.001	0.005
		Severe disability	− 0.011	0.013	− 0.009	0.016	0.002	0.010	0.014	0.009*
	Time *t* − 1	Benefit	− 0.101	0.010***	− 0.044	0.012***	− 0.066	0.011***	− 0.013	0.007*

Source: Authors’ elaborations on EU-SILC data

We control for the set of covariates described in the section “Data and variables” including regional and year dummies. *p < .10, **p < .05, ***p < .01

Looking at the top panel of Table 3, we note that past poverty status reduces the probability of full work intensity in all countries explored. The AMEs are statistically significant for France and the UK, with AMEs of −5.5 pp. and −5.7 pp, respectively. In Germany and Italy, past poverty is not statistically significant, whereas initial poverty status significantly decreases full work intensity (by −5.1 pp. and −2.8 pp., respectively). In general, this suggests that feedback effects are at work. The fact that previous poverty status reduced full WI constitutes a violation of the strict exogeneity assumption and can be interpreted as evidence for the disincentive effects of low income on labor market attachment/participation [6].

As far as state dependence in WI is concerned, we see that this is an issue in all countries explored, but with different magnitudes. On the one hand, this signals a strong state-dependence effect in employment behavior, i.e., even after controlling for other characteristics, employment in one period led to higher employment probabilities in future periods. On the other hand, this might reflect the state-dependence effects of unemployment found in other studies (such as [33] for Germany and [2] for the UK). Persistence in full WI, for example, ranges from 49.2 pp. in Italy to 68 pp. in France. For the other ranges of WI, we also find positive and significant AMEs. Nonetheless, as expected, the magnitude of such effects reduces with the intensity of WI.

Finally, we examine the relationship between disability and WI. From Table 3, we note that for all countries, there is a negative association between current disability and WI that increases with the severity of the disability. Severe disability is particularly detrimental in Southern Europe (−6.4 pp. for Italy), while the estimated AME is −3.4 pp for Germany. If we consider severe disability in the previous period, we find that the detrimental effect of severe disability persists only in Germany (−3.5 pp.). The negative effect of the presence of a disabled person in the household (especially someone with severe activity limitations) on household WI might be due to disabled persons not working/reducing their work and because other household members may need to reduce their work to care for the person with disabilities.

Interestingly, looking at the effect of disability cash benefits (previous year), we note that these are negatively associated with work intensity in all countries explored. The AMEs range from –1.3 pp. in Italy to –10.1 pp. in the UK. The different magnitudes may reflect country differences in terms of how disability benefits work, such as the minimum level of incapacity for work. For example, in countries where legislation is relatively more restrictive (such as Italy), the average effect on work intensity should be smaller compared to countries with less-restrictive regulations (e.g., the UK). For details on country differences regarding how disability benefits work in each country, see MISSOC data.

### Disability

The results for the disability equation are reported in Table 4. Note that in this equation, we also include lagged poverty experience and lagged WI, capturing possible feedback effects from poverty and WI to disability. Previous poverty status is positively and significantly associated with disability only in Germany (+ 2.1 pp.). However, we found a positive association between initial poverty status and severe disability in the UK and Italy (+ 2.8 pp. and + 1 pp., respectively). This finding is in line with the work of Jenkins and Rigg [22], who found that in the UK, the average income of disabled working-age persons falls sharply with the onset of disability but subsequently recovers. Further, this suggests the presence of feedback effects from past poverty to disability. As for the possible feedback effects of WI, we see that this is an issue in France, where past full WI increases the probability of having a severely disabled household member by 2.8 pp.

**Table 4:** Disability equation: outcome of severe disability

			UK		Germany		France		Italy	
			AME	s.e	AME	s.e	AME	s.e	AME	s.e
Poverty	Time *t* − 1		0.014	0.010	0.021	0.009**	0.003	0.008	− 0.007	0.007
	Time 1		0.028	0.010***	− 0.003	0.009	0.012	0.008	0.010	0.005*
Work intensity	Time *t* − 1	0 < WI ≤ 0.5	− 0.025	0.017	0.005	0.014	0.007	0.011	0.002	0.007
		0.5 < WI < 1	− 0.042	0.016***	− 0.012	0.012	0.000	0.011	− 0.006	0.007
		WI = 1	0.002	0.019	0.001	0.016	0.028	0.013**	0.010	0.009
	Time 1	0 < WI ≤ 0.5	− 0.025	0.018	− 0.003	0.015	− 0.014	0.013	− 0.014	0.008*
		0.5 < WI < 1	− 0.004	0.016	− 0.010	0.013	− 0.011	0.013	− 0.003	0.007
		WI = 1	− 0.028	0.019	− 0.013	0.016	− 0.023	0.015	− 0.011	0.008
Disability	Time *t* − 1	Disability	0.019	0.007***	0.011	0.006*	0.011	0.005**	0.012	0.003***
		Severe disability	0.011	0.010	0.024	0.010**	0.025	0.008***	0.009	0.005*
	Time 1	Disability	0.200	0.006***	0.131	0.004***	0.151	0.003***	0.119	0.003***
		Severe disability	0.298	0.010***	0.236	0.012***	0.233	0.006***	0.163	0.003***
	Time *t* − 1	Benefit	0.049	0.007***	0.057	0.006***	0.054	0.008***	0.048	0.004***

Source: Authors’ elaborations on EU-SILC data

We control for the set of covariates described in the section “Data and variables”, including regional and year dummies. *p < .10, **p < .05, ***p < .01

Finally, we find a relatively small state dependence, with the highest AMEs in Germany (+ 2.4 pp.). Notably, we see that in the long-term persistence in severe disability is much stronger (ranging from + 16.3 pp. for Italy to + 29.8 pp. for the UK).

As stated above, to avoid reverse causality, we considered the previous year’s disability cash benefits, also assuming that disability might be a varying condition. From Table 4, we note that the presence of disability cash benefits is positively associated with disability; this may partly be due to justification bias, which refers to individuals/respondents overstating their level of disability to justify welfare receipt (see, for instance, [7]).

### Further results and supplementary analyses

In this section, we discuss the effects of other covariates common to the three equations, the role of random effects, additional evidence on the role of extra costs, and alternative indicators of poverty. With regard to the rest of the covariates (for details, see the section “The econometric approach”),^10^ it is necessary to stress the importance of higher education (tertiary educational attainment level) for all of the phenomena examined and in all explored countries. Higher education is a protective factor against poverty, in addition to being positively associated with work intensity/labor market participation, while it exerts a less clear role on (severe) disability. In particular, one of the key findings is that households with a low-educated head of household are particularly disadvantaged in terms of poverty and work intensity. These effects might be due to institutional frictions preventing disadvantaged households from accessing critical public services, e.g., low-educated individuals may have difficulties dealing with demanding administrative procedures unless some type of public support is provided. Moreover, we find that female heads of household are more likely to be poor in all countries examined. For the other phenomena, instead, we do not find a clear role of gender. All in all, the findings from the three equations suggest that state dependence is an important issue for all phenomena investigated. The results also show the differing significance of the feedback effects considered. We find feedback effects of poverty on WI in all countries investigated, and the results show that previous poverty is associated with current disability in all countries except France.

We also find that the random-effect terms of each equation are relevant in terms of magnitude and are sometimes correlated two by two (see Table 5), thus indicating possible endogeneity issues because of selection due to shared unobservable factors. The correlation terms show the expected sign when statistically significant. In particular, *ρ*_*pw*_ and *ρ*_*wd*_ are negative. On the one hand, this suggests that unobservable factors that increase the risk of poverty are actually detrimental to the probability of experiencing full work intensity. On the other hand, unobservable factors that increase the probability of experiencing full work intensity decrease the probability of having a person with severe disability in the household.

**Table 5:** Random-effects parameters and residuals

Random-effects parameters	UK		Germany		France		Italy	
	Estimate	s.e	Estimate	s.e	Estimate	s.e	Estimate	s.e
Standard deviations								
* μ*	0.734	0.075	0.870	0.090	0.992	0.073	0.934	0.071
* ϵ*	0.571	0.045	0.610	0.050	0.536	0.037	0.450	0.042
* κ*	0.927	0.054	0.955	0.063	0.954	0.043	1.033	0.054
Cross-equation correlations								
* ρpw*	− 0.231	0.123	− 0.335	0.105	− 0.033	0.099	− 0.235	0.097
* ρpd*	0.064	0.091	− 0.005	0.094	− 0.109	0.065	0.061	0.078
* ρwd*	− 0.182	0.084	− 0.088	0.095	− 0.146	0.072	− 0.082	0.078
Residuals								
Standard deviations								
* u*	1.000		1.000		1.000		1.000	
* v*	1.000		1.000		1.000		1.000	
* ε*	1.000		1.000		1.000		1.000	
Cross-equation correlations								
Corr *(u, v)*	− 0.096	0.147	− 0.285	0.104	− 0.104	0.183	0.005	0.094
Corr *(u, ε)*	0.147	0.077	0.111	0.112	0.165	0.074	− 0.082	0.093
Corr *(v, ε)*	0.107	0.054	− 0.113	0.065	0.181	0.049	0.144	0.044

Source: Authors’ elaborations on EU-SILC data

As a supplementary analysis, we run our model without accounting for the extra costs of disability (Appendix Tables 7, 8, 9) We find that the association between severe disability and poverty is smaller and less statistically significant. This confirms the relevance of considering the effect of the extra costs of disability.

Finally, we estimate our model considering severe material deprivation instead of poverty (Appendix Tables 10, 11, 12). In contrast to poverty, severe material deprivation is a non-monetary indicator based on the lack of financial resources. Our findings suggest that state dependence in severe material deprivation is an issue only for Germany and Italy (Table 10), while for poverty, we find state dependence in all explored countries. For WI and severe disability (Tables 11, 12), we find state dependence in line with the results for poverty. As for feedback effects, the results are more mixed across countries compared to those for poverty.

### Reflections on cross-country differences

We offer some reflections on the heterogeneity across countries in our findings in terms of the complex relationship between poverty, work intensity, and disability. These differences suggest different policies implications for each country.

We found heterogeneity in the effects of disability on poverty that emerge across countries. First, we note a divergence from descriptive evidence based on official poverty rates (Fig. 1) and those that emerge from quantitative analysis (Table 2). The case of Germany is quite illustrative, as it shows the greatest differences in official poverty rates by activity limitation level, while the estimated impact of disability on poverty is the smallest among the analyzed countries. The opposite emerges in Italy. Considering that the official poverty rates do not account for the extra costs of disability, the mentioned divergence possibly highlights the importance of in-kind transfers to mitigate the disadvantage of households with disabled members. This speculation is reinforced by focusing on the level of benefits for social exclusion, disability, and sickness/health care functions (Table 6).^11^ The positive association between disability and poverty is weaker for Germany, where in-kind disability and sickness/health care benefits are significantly above the EU average. In contrast, the positive association is stronger where such benefits are significantly below the EU average, such as in the UK and Italy. The link with disability cash benefits is less clear (Table 6).

Thus, policies oriented to the generosity of disability and health care benefits^12^ may help mitigating the detrimental effects of disability on the risk of poverty. However, a more functional system of disability benefits may sustain people with disabilities and their households by also facilitating the labor market integration of the former and reducing informal care provided within the family, with positive consequences for household labor supply and income formation (e.g., [35]). An effective combination of policies for disability benefit schemes and labor market institutions, namely the availability of flexible working hours and part-time opportunities, may help reconcile market and non-market work (including caring activities for disabled members), with positive effects on labor supply and household income.

Notably, the countries where social exclusion benefits are higher are also those where the detrimental effect of disability on poverty is greater. Thus, disability benefits appear to be more effective than those aimed at social exclusion for the incomes of households with disabled members.

Looking at social protection benefits (all functions), we struggle to identify a clear protective role against poverty. For example, despite Italy showing relatively high levels of cash and in-kind social protection benefits (many of these for older people), the impact of disability on poverty is relevant, while in Germany, the impact is small despite social protection benefits being below the EU average. This might suggest that the expenditure across different functions of social protection is not highly effective in alleviating the detrimental effect of disability on poverty.

We also note that the estimated impact of disability on poverty changes depending on whether we account for feedback effects or not and, particularly, when integrating the role of work intensity in the disability–poverty relationship. AMEs obtained from the benchmark model (Table 2) for all countries are smaller than those obtained from the single poverty equation (Table 13), with the exception of Italy where disability effects are rather consistent across the specification. This allows us to highlight how the largest disability effects significantly depend on the role of work intensity. Despite work intensity greatly contributing to reducing poverty in Italy, its insurance role is weakened by the detrimental effect disability exerts on work intensity. This can be associated with the widespread use of informal care, which is usually provided by female family members. This stresses a possible role of formal care responsibilities and labor market institutions to avoid females being constrained to stay out of the labor force and the associated detrimental impact for disability effects on poverty via work intensity.

Eurostat data on population by care responsibilities and labor status^13^ across countries in 2018 indeed show an important gender gap in care responsibilities. On average, in all countries, explored women both in and out the labor force are more likely to take on caring roles (for incapacitated relatives) than men. These additional data might help explain the relatively higher rates of poverty (by level of disability), as women are often constrained to stay outside of the labor force in order to care for disabled household members.

Our results for the WI equation show that severe disability is detrimental to labor supply in all countries explored, and especially in Italy and France (Table 3). It is mainly in these countries that there is a need for policy interventions aimed at reducing both the direct and indirect effects of disability. For the former, the integration of disabled individuals in the labor market may benefit from more flexible working conditions (in terms of working hours and workplace, for instance). For the latter, supporting the caring activities of other household members is crucial. This appears particularly important in Italy, where females remaining outside of the labor force due to caring activities is common. Notably, we also find a significant detrimental role of disability benefits on WI in all countries, and especially in the UK. This might suggest the need for policy interventions to redesign the disability benefits.

Finally, going back to the negative association estimated between disability benefits and poverty, we need to be aware of the fact the provision of such benefits was affected by the austerity measures adopted in the context of the Great Recession. These interventions determined significant cuts in public spending, in many countries including that targeted at people with disabilities (especially the UK and Italy, among those investigated; for details, see [36]). Therefore, in considering the protective role of benefits and their current level (Table 6) and the mentioned cuts, an increasing investment in such policy alleviation measures is advisable, in particular where public cuts were particularly severe.

## Concluding remarks

This paper offers a framework for the study of the relationship between poverty, work, and disability. We analyze four major Western European countries using data for 2015–2018 and a dynamic trivariate panel data model that accounts for state dependence, correlated random effects, endogenous initial conditions, and feedback effects. In addition, we account for the extra costs of disability by adopting a disability-adjusted equivalence scale to correct household income.

We find a positive and statistically significant association between the risk of poverty and the severity of disability. The effects are relatively consistent and sizeable across countries, despite the mitigating role played by disability cash benefits. The strongest magnitude of the effect of severe disability on poverty is found in the UK, whereas the weakest emerges in Germany. The relatively smaller effect we found in Germany might be due to institutions, i.e., relatively high provision of in-kind transfers to mitigate the disadvantage of persons with disabilities, as well as to more effectively promoted reconciliation policies and a more inclusive labor market for females.

Cross-country differences may reflect different social and disability policies, including the importance and effectiveness of in-kind disability transfers. In addition, current work intensity plays an important mediating role in the disability–poverty connection, with different strengths and timing. Despite work intensity greatly contributing to reducing poverty in Italy, its insurance role is weakened by the detrimental effect disability exerts on work intensity. This may be partly due to the widespread use of informal care, which is usually provided by female family members, especially in Southern Europe.

Our analysis reveals the presence of feedback effects from past poverty to work intensity and from past work intensity to disability. Feedback effects from past poverty to disability are present in the UK, Germany, and Italy. All in all, this suggests that disadvantageous positions reinforce one another, thus favoring income and health polarization over time in societies.

We also find evidence of sizeable genuine state dependence in all analyzed phenomena. Poverty state dependence is stronger in Italy and weaker in the UK, France, and especially Germany, while persistence tends to increase over time in all countries. Work intensity is characterized by a sizeable degree of state dependence, with differences across countries. Disability state dependence is relatively small in all countries. The amount of state dependence in full work intensity appears to be lower in countries characterized by relatively greater labor market flexibility. These results highlight the relevance of measures aimed at lifting individuals out of poverty and low work intensity to avoid disadvantage persisting over time. All in all, the heterogeneity uncovered across the countries explored suggests that country-specific policy interventions need to be designed and implemented for each nation.

The analysis presents some limitations, some of which are related to the unavailability of more complete data. Despite the longitudinal EU-SILC dataset being a valuable source of information for this type of analysis, there is space for improvement. Administrative data would help to identify disability through medical certification, which may mitigate the issue of the subjective nature of self-reported information. In addition, contextual data describing the external environment would help to account for local heterogeneity in physical and cultural barriers that increase the limitations of people with disabilities. The EU-SILC data include some environmental variables, but only in the cross-sectional files. In addition, more precise data on time allocation would allow us to identify caring responsibilities within the household and to better qualify the indirect effects of disability on the labor supply of family members. Finally, a longer panel would also help to more effectively identify state dependence and characterize long-term issues. The availability of more detailed information should be the basis for future research along the lines described above.

## Data Availability

Restrictions apply to the availability of the data used under license from EUROSTAT for this study. Data are available from the authors upon reasonable request and with permission of EUROSTAT only.

## Appendix

See Tables

**Table 6:** Level of benefits for social protection by country, with respect to the EU average in the 2015–2018 period

Indicator	UK	Germany	France	Italy
Social protection benefits (all functions)—total	Aligned	Significantly below	Significantly above	Above
Social protection benefits (all functions)—cash	Above	Significantly below	Significantly above	Above
Social protection benefits (all functions)—in kind	Significantly below	Slightly below	Significantly above	Slightly above
Disability benefits—total	Slightly below	Significantly above	Slightly below	Below
Disability benefits—cash	Aligned	Above	Slightly below	Aligned
Disability benefits—in kind	Significantly below	Significantly above	Significantly above	Significantly below
Sickness/health care benefits total	Aligned	Significantly above	Above	Below
Sickness/health care benefits—cash	Significantly below	Aligned	Below	Significantly below
Sickness/health care benefits—in kind	Slightly above	Significantly above	Above	Below
Social exclusion benefits—total	Aligned	Significantly below	Significantly above	Above
Social exclusion benefits—cash	Above	Significantly below	Significantly above	Above
Social exclusion benefits—in kind	Significantly below	Aligned	Significantly above	Slightly above

We adopt the following definitions: “aligned” if within + /– 5% of the EU average in 2015–2018; “slightly above/below” if within + /– 5–15% of the EU average; “above/below” if within + /– 15/30% of the EU average; “significantly above/below” if over + /– 30% of the EU average

Source: Authors elaborations on Eurostat data

6,

**Table 7:** Poverty equation: no extra costs for disability

			UK		Germany		France		Italy	
			AME	s.e	AME	s.e	AME	s.e	AME	s.e
Poverty	Time *t* − 1		0.037	0.010***	0.067	0.008***	0.059	0.006***	0.074	0.009***
	Time 1		0.127	0.007***	0.096	0.007***	0.089	0.005***	0.146	0.005***
Work intensity	Time *t*	0 < WI ≤ 0.5	− 0.022	0.030	− 0.006	0.009	− 0.096	0.019***	− 0.016	0.022
		0.5 < WI < 1	− 0.050	0.052	0.007	0.005	− 0.174	0.014***	− 0.094	0.041**
		WI = 1	− 0.066	0.073	0.045	0.003***	− 0.209	0.014***	− 0.121	0.057**
	Time *t* − 1	0 < WI ≤ 0.5	0.026	0.016*	− 0.037	0.023	− 0.006	0.010	0.016	0.008**
		0.5 < WI < 1	− 0.003	0.019	− 0.070	0.020***	− 0.008	0.009	0.001	0.011
		WI = 1	0.007	0.030	− 0.091	0.021***	0.004	0.010	0.004	0.018
	Time 1	0 < WI ≤ 0.5	− 0.039	0.026	− 0.021	0.021	0.015	0.008*	− 0.025	0.009***
		0.5 < WI < 1	− 0.067	0.028**	− 0.049	0.018***	0.019	0.007***	− 0.012	0.010
		WI = 1	− 0.100	0.033***	− 0.078	0.019***	0.018	0.008**	− 0.006	0.015
Disability	Time *t*	Disability	0.014	0.007**	0.004	0.010	0.006	0.015	− 0.006	0.004
		Severe disability	0.035	0.013***	0.009	0.001***	− 0.004	0.000***	− 0.012	0.010
	Time *t* − 1	Disability	− 0.001	0.007	0.005	0.008	0.006	0.006	0.000	0.004
		Severe disability	− 0.015	0.009*	0.002	0.010	0.014	0.007*	− 0.019	0.008**
	Time 1	Disability	− 0.002	0.008	0.000	0.008	− 0.004	0.005	− 0.010	0.005**
		Severe disability	0.001	0.011	0.016	0.012	− 0.008	0.007	− 0.001	0.008
	Time *t* − 1	Benefit	− 0.052	0.010***	− 0.023	0.008***	− 0.038	0.008***	− 0.021	0.006***

Source: Authors’ elaborations on EU-SILC data

7,

**Table 8:** Work intensity equation (WI = 1): no extra costs for disability

			UK		Germany		France		Italy	
			AME	s.e	AME	s.e	AME	s.e	AME	s.e
Poverty	Time *t* − 1		− 0.027	0.013**	− 0.010	0.015	− 0.047	0.012***	− 0.003	0.010
	Time 1		− 0.019	0.012	− 0.034	0.014**	− 0.020	0.010*	− 0.028	0.009***
Work intensity	Time *t* − 1	0 < WI ≤ 0.5	0.053	0.009***	0.063	0.013***	0.039	0.006***	0.043	0.007***
		0.5 < WI < 1	0.192	0.007***	0.213	0.010***	0.203	0.007***	0.171	0.008***
		WI = 1	0.499	0.019***	0.531	0.017***	0.679	0.011***	0.494	0.022***
	Time 1	0 < WI ≤ 0.5	0.111	0.015***	0.073	0.017***	0.101	0.016***	0.061	0.010***
		0.5 < WI < 1	0.186	0.011***	0.183	0.013***	0.174	0.014***	0.154	0.011***
		WI = 1	0.389	0.023***	0.461	0.022***	0.336	0.020***	0.339	0.024***
Disability	Time *t*	Disability	− 0.017	0.007**	− 0.029	0.008***	− 0.003	0.006	− 0.015	0.004***
		Severe disability	− 0.047	0.013***	− 0.036	0.017**	− 0.059	0.011***	− 0.062	0.010***
	Time *t* − 1	Disability	0.004	0.008	− 0.001	0.010	− 0.011	0.007*	0.005	0.005
		Severe disability	− 0.001	0.012	− 0.033	0.016**	− 0.013	0.010	− 0.013	0.009
	Time 1	Disability	0.004	0.009	0.002	0.010	0.001	0.007	− 0.001	0.005
		Severe disability	− 0.013	0.013	− 0.017	0.016	− 0.001	0.009	0.011	0.009
	Time *t* − 1	Benefit	− 0.100	0.010***	− 0.042	0.012***	− 0.065	0.011***	− 0.013	0.007*

Source: Authors’ elaborations on EU-SILC data

8,

**Table 9:** Disability equation (severe disability): no extra costs for disability

			UK		Germany		France		Italy	
			AME	s.e	AME	s.e	AME	s.e	AME	s.e
Poverty	Time *t* − 1		0.012	0.010	0.012	0.013	0.002	0.021	− 0.008	0.007
	Time 1		0.016	0.010*	0.002	0.011	0.004	0.016	0.013	0.007**
Work intensity	Time *t* − 1	0 < WI ≤ 0.5	− 0.026	0.017	0.006	0.016	0.008	0.013	0.002	0.007
		0.5 < WI < 1	− 0.042	0.016***	− 0.012	0.012	0.001	0.012	− 0.006	0.007
		WI = 1	0.004	0.019	0.002	0.013	0.029	0.013**	0.010	0.009
	Time 1	0 < WI ≤ 0.5	− 0.027	0.018	− 0.005	0.016	− 0.017	0.014	− 0.014	0.008*
		0.5 < WI < 1	− 0.012	0.016	− 0.012	0.013	− 0.016	0.013	− 0.002	0.007
		WI = 1	− 0.037	0.019*	− 0.016	0.014	− 0.028	0.014**	− 0.010	0.008
Disability	Time *t* − 1	Disability	0.019	0.008**	0.011	0.007	0.011	0.007	0.012	0.003***
		Severe disability	0.013	0.010	0.028	0.011***	0.025	0.009***	0.008	0.005
	Time 1	Disability	0.197	0.006***	0.131	0.004***	0.150	0.003***	0.119	0.003***
		Severe disability	0.303	0.010***	0.235	0.012***	0.235	0.006***	0.164	0.003***
	Time *t* − 1	Benefit	0.048	0.008***	0.057	0.006***	0.053	0.008***	0.049	0.004***

Source: Authors’ elaborations on EU-SILC data

9,

**Table 10:** Severe material deprivation (MD) equation

			UK		Germany		France		Italy	
			AME	s.e	AME	s.e	AME	s.e	AME	s.e
Severe MD	Time *t* − 1		0.006	0.005	0.010	0.006*	0.003	0.003	0.016	0.007*
	Time 1		0.045	0.004***	0.037	0.004***	0.040	0.003***	0.052	0.005***
Work intensity	Time *t*	0 < WI ≤ 0.5	− 0.020	0.011*	− 0.009	0.006*	− 0.008	0.004	− 0.026	0.015*
		0.5 < WI < 1	− 0.024	0.015	− 0.006	0.007	− 0.004	0.005	− 0.053	0.023*
		WI = 1	− 0.039	0.017*	0.001	0.012	− 0.004	0.008	− 0.070	0.029*
	Time *t* − 1	0 < WI ≤ 0.5	0.008	0.007	0.005	0.006	− 0.002	0.004	0.015	0.007*
		0.5 < WI < 1	0.015	0.007*	0.001	0.005	− 0.002	0.004	0.008	0.007
		WI = 1	0.019	0.010*	− 0.001	0.006	− 0.007	0.005	0.035	0.013***
	Time 1	0 < WI ≤ 0.5	0.004	0.010	− 0.014	0.009	0.008	0.004*	− 0.001	0.009
		0.5 < WI < 1	− 0.013	0.008*	− 0.014	0.010	0.006	0.004	− 0.004	0.009
		WI = 1	− 0.009	0.011	− 0.021	0.011*	− 0.001	0.004	− 0.020	0.012*
Disability	Time *t*	Disability	0.002	0.005	0.001	0.006	0.005	0.004	0.024	0.012*
		Severe disability	0.025	0.012*	0.005	0.014	0.009	0.007	0.046	0.027*
	Time *t* − 1	Disability	0.004	0.005	− 0.002	0.003	− 0.001	0.002	0.013	0.005***
		Severe disability	0.005	0.006	0.002	0.005	− 0.001	0.003	0.002	0.007
	Time 1	Disability	− 0.005	0.006	0.008	0.005	0.005	0.003*	− 0.006	0.006
		Severe disability	− 0.002	0.008	0.005	0.008	0.011	0.005*	− 0.013	0.008
	Time *t* − 1	Disability benefit	− 0.004	0.004	− 0.006	0.004*	− 0.003	0.003	0.001	0.005

Source: Authors’ elaborations on EU-SILC data

10,

**Table 11:** Work intensity equation: outcome WI = 1 (with severe MD indicator)

			UK		Germany		France		Italy	
			AME	s.e	AME	s.e	AME	s.e	AME	s.e
Severe MD	Time *t* − 1		− 0.030	0.019	− 0.036	0.025	− 0.052	0.015***	0.023	0.010*
	Time 1		− 0.026	0.018	− 0.039	0.024	− 0.016	0.014	− 0.031	0.008***
Work intensity	Time *t* − 1	0 < WI ≤ 0.5	0.054	0.009***	0.060	0.011***	0.038	0.005***	0.042	0.007***
		0.5 < WI < 1	0.195	0.007***	0.213	0.008***	0.205	0.007***	0.172	0.008***
		WI = 1	0.496	0.019***	0.535	0.024***	0.680	0.014***	0.495	0.022***
	Time 1	0 < WI ≤ 0.5	0.106	0.015***	0.081	0.015***	0.110	0.015***	0.063	0.009***
		0.5 < WI < 1	0.189	0.011***	0.190	0.013***	0.189	0.015***	0.160	0.011***
		WI = 1	0.401	0.023***	0.467	0.031***	0.363	0.026***	0.353	0.024***
Disability	Time *t*	Disability	− 0.014	0.020	− 0.022	0.020	− 0.006	0.017	− 0.003	0.013
		Severe disability	− 0.030	0.036	− 0.056	0.035	− 0.028	0.031	− 0.018	0.022
	Time *t* − 1	Disability	0.008	0.011	0.002	0.011	− 0.005	0.007	0.019	0.006***
		Severe disability	− 0.010	0.016	− 0.029	0.019	− 0.021	0.012*	0.000	0.010
	Time 1	Disability	0.020	0.020	− 0.001	0.014	− 0.005	0.010	− 0.015	0.007*
		Severe disability	0.020	0.020**	− 0.011	0.023	− 0.010	0.016	− 0.011	0.011
	Time *t* − 1	Disability benefit	− 0.097	0.011***	− 0.040	0.013***	− 0.064	0.012***	− 0.015	0.007*

Source: Authors’ elaborations on EU-SILC data

11,

**Table 12:** Disability equation: outcome of severe disability (with severe MD indicator)

			UK		Germany		France		Italy	
			AME	s.e	AME	s.e	AME	s.e	AME	s.e
Severe MD	Time *t* − 1		0.005	0.013	0.013	0.010	− 0.004	0.009	− 0.002	0.004
	Time 1		0.041	0.013***	0.024	0.010*	0.036	0.009***	− 0.003	0.004
Work intensity	Time *t* − 1	0 < WI ≤ 0.5	− 0.006	0.016	0.004	0.010	0.008	0.009	0.008	0.004*
		0.5 < WI < 1	− 0.028	0.015*	− 0.011	0.009	− 0.001	0.008	0.007	0.004*
		WI = 1	− 0.043	0.018*	− 0.021	0.011*	− 0.005	0.010	0.014	0.005*
	Time 1	0 < WI ≤ 0.5	0.002	0.016	0.003	0.010	− 0.010	0.010	− 0.015	0.005**
		0.5 < WI < 1	− 0.014	0.014	− 0.001	0.009	− 0.010	0.010	− 0.014	0.005***
		WI = 1	− 0.018	0.017	− 0.005	0.010	− 0.021	0.011*	− 0.023	0.006***
Disability	Time *t* − 1	Disability	0.053	0.008***	0.028	0.005***	0.023	0.004***	0.012	0.003***
		Severe disability	0.085	0.015***	0.052	0.010***	0.071	0.010***	0.031	0.006***
	Time 1	Disability	0.131	0.009***	0.083	0.006***	0.109	0.006***	0.047	0.003***
		Severe disability	0.343	0.025***	0.329	0.031***	0.285	0.018***	0.127	0.009***
	Time *t* − 1	Disability benefit	0.059	0.007***	0.055	0.005***	0.071	0.007***	0.037	0.003***

Source: Authors’ elaborations on EU-SILC data

12 and

**Table 13:** Single Poverty equation

			UK		Germany		France		Italy	
			AME	s.e	AME	s.e	AME	s.e	AME	s.e
Poverty	Time *t* − 1		0.048	0.011***	0.035	0.009***	0.043	0.007***	0.077	0.009***
	Time 1		0.113	0.008***	0.102	0.006***	0.093	0.005***	0.149	0.005***
Work intensity	Time *t*	0 < WI ≤ 0.5	− 0.081	0.021***	− 0.044	0.015***	− 0.063	0.014***	− 0.021	0.011*
		0.5 < WI < 1	− 0.158	0.020***	− 0.083	0.013***	− 0.116	0.014***	− 0.107	0.010***
		WI = 1	− 0.199	0.021***	− 0.096	0.014***	− 0.139	0.014***	− 0.153	0.011***
	Time *t* − 1	0 < WI ≤ 0.5	0.015	0.015	0.002	0.012	− 0.017	0.009*	0.011	0.008
		0.5 < WI < 1	0.002	0.012	− 0.004	0.010	− 0.017	0.008**	− 0.011	0.007
		WI = 1	0.003	0.014	− 0.015	0.010	− 0.017	0.009*	− 0.016	0.008*
	Time 1	0 < WI ≤ 0.5	0.000	0.017	− 0.036	0.013***	0.017	0.008**	− 0.013	0.008
		0.5 < WI < 1	− 0.016	0.014	− 0.028	0.012**	0.016	0.007**	− 0.006	0.007
		WI = 1	− 0.024	0.015	− 0.023	0.013*	0.012	0.008	0.017	0.009**
Disability	Time *t*	Disability	0.004	0.006	0.000	0.005	0.003	0.003	0.001	0.004
		Severe disability	0.173	0.013***	0.110	0.014***	0.124	0.009***	0.118	0.011***
	Time *t* − 1	Disability	− 0.009	0.007	− 0.002	0.006	0.005	0.004	− 0.006	0.004
		Severe disability	− 0.017	0.009**	− 0.013	0.008*	− 0.009	0.006	− 0.050	0.007***
	Time 1	Disability	0.005	0.008	0.006	0.007	− 0.006	0.005	− 0.007	0.005
		Severe disability	− 0.017	0.009*	− 0.005	0.009	− 0.017	0.006***	− 0.025	0.007***
	Time *t* − 1	Benefit	− 0.048	0.008***	− 0.009	0.007	− 0.031	0.007***	− 0.027	0.006***

Source: Authors’ elaborations on EU-SILC data

We control for the set of covariates described in the section “Data and variables”, including regional and year dummies. **p* < .10, ***p* < .05, ****p* < .01

^13^

## Footnotes

1 For details on methodologies for income-poverty measurement, see [15].

2 Among sources of endogeneity, we also consider the potential correlation of unobservable factors affecting each process.

3 According to Eurostat data for the year 2012, the share of people with disabilities ranges from 13.6% in France to approximately 21% in Germany. Figures available online at http://appsso.eurostat.ec.europa.eu/nui/show.do.

4 Modified OECD scale: 1 + 0.5(*N*_*A*_ - 1) + 0.3 *N*_*CH*_; disability-corrected equivalence scale: 1.56[1 + 0.5(*N*_*D*_ - 1)] + 0.5(*N*_*A*_ - *N*_*D*_) + 0.3 *N*_*CH*_, where *N*_*A*_, *N*_*CH*_, and *N*_*D*_ are the number of adults, children, and disabled persons in the household, respectively. We are aware of the fact that extra costs may vary across countries. However, according to extra-cost estimates (e.g., [32]), the differences across the countries we examine are relatively small.

5 For a detailed explanation, see the Eurostat website: https://ec.europa.eu/eurostat/statistics-explained/index.php?title=Glossary:Persons_living_in_households_with_low_work_intensity.

6 The model is estimated using STATA’s *cmp* command, written by Roodman [44].

7 The same author stressed that using this model is helpful for treating attrition, as the CML estimator allows a different attrition probability depending on the initial value of each outcome. This ensures that attrition is implicitly taken into account.

8 We also conduct a heterogeneity analysis by estimating our model separately by gender of the head of household, by age (head of household below 35 years), and by urban setting. For the sake of brevity, we do not report these results here. These are available upon request.

9 The results for the remaining outcomes for work intensity and disability are available upon request.

10 For the sake of brevity, the relevant tables are available upon request.

11 Figures available online at https://ec.europa.eu/eurostat/databrowser/view/SPR_EXP_PPSH__custom_1478214/default/table.

12 There are differences in disability benefits across countries in terms of basic principles, fields of application, exemptions, and eligibility conditions (e.g., the minimum level of incapacity for work) (MISSOC data, October 2023).

13 Figures available online at https://ec.europa.eu/eurostat/databrowser/view/LFSO_18CRESLS__custom_1481561/default/table.
